# Volunteering for Health Services in the Middle Part of Ghana: In Whose Interest?

**DOI:** 10.15171/ijhpm.2018.38

**Published:** 2018-05-05

**Authors:** Samuel Afari-Asiedu, Kwaku Poku Asante, Kodjo Senah, Martha Ali Abdulai, Stephen Afranie, Emmanuel Mahama, Edward Apraku Anane, Mahama Abukari, Martin Luther Darko, Lawrence G. Febir, Seth Owusu-Agyei

**Affiliations:** ^1^Kintampo Health Research Centre, Ghana Health Service, Kintampo, Ghana.; ^2^Department of Sociology, School of Social Science, University of Ghana, Accra, Ghana.; ^3^Institute of Health Research, University of Health and Allied Sciences, Ho, Ghana.

**Keywords:** Community Volunteers’ Motivation, Community Volunteers’ Retention, Community Volunteers’ Satisfaction, Monetary Incentives, Non-monetary Incentives

## Abstract

**Background:** In many developing countries like Ghana, community volunteers assist in the provision of certain health services to rural and hard-to-reach communities. This study examined factors that influence the motivation and retention of community-based volunteers supporting with work on health-related activities at the community level in Ghana.

**Methods:** Using a sequential mixed-method design, a cross-sectional survey was carried out among 205 selected community-based volunteers in Kintampo North Municipality (KNM) and Kintampo South District (KSD) of Ghana etween December, 2014 and February, 2015. Qualitative interviews, including 12 in-depth interviews (IDIs) among health workers and community opinion leaders and 2 focus group discussion (FGD) sessions with volunteers were conducted.

**Results:** Personal interest (32.7%) and community leaders’ selection of volunteers (30.2%) were key initial reasons for volunteering. Monetary incentives such as allowance for extra duty (88.8%) and per diem (49.3%) and non-monetary incentives such as T-shirts/bags (45.4 %), food during training (52.7%), community recognition, social prestige and preferential treatment at health facilities were the facilitators of volunteers’ retention. There was a weak evidence (P=.051) to suggest that per diem for their travels is a reason for volunteers’ satisfaction.

**Conclusion:** Community-based volunteers’ motivation and retention were influenced by their personal interest in the form of recognition by community members and health workers, community leaders’ selection and other nonmonetary incentives. Volunteers were motivated by extra-duty allowance but not per diems paid for accommodation and feeding when they travel. Organizations that engage community volunteers are encouraged to strengthen the selection of volunteers in collaboration with community leaders, and to provide both non-monetary and monetary incentives to motivate volunteers.

## Background

The shortage of health workers is universally acknowledged as a key development challenge, a barrier to strengthening health systems and improving the prospects for achieving universal health coverage.^1^ In 2006, the global health worker shortage stood at 4.3 million.^1^ The figure shot up to 7.2 million in 2013 and estimated to go up to 12.9 million by 2035.^2^ In Ghana, in spite of the increase in the number of trained health workers in recent past, there is still inadequate health workers particularly in the rural communities where they are needed most.^3^ It is therefore evident that if nothing is done to address the shortage of health workforce now, it would have serious implications for the health of billions of people across all regions of the world.^2^

Calls for innovative and effective approaches that address the workforce challenge have resulted in a resurgence of various countries interest in and attention to community-based volunteers (CBVs) programmes that is expected to extend healthcare coverage and key interventions to hard-to-reach communities.^4,5^ In Africa, CBVs mostly support health workforce where they are insufficient because of lack of incentives to attract and retain them.

CBVs are lay members of communities who work in association with the local healthcare system in mostly rural but also some urban settings. They usually share ethnicity, language, economic status and life experiences with the community members they serve.^5^ CBVs play vital roles in linking diverse and hard to reach population to health and social service systems. CBVs serve as a bridge between professional health staff and the community. They also help communities identify and address their own health needs, as such they are increasingly engaged by Ministries of Health to extend health services to local settings.^5,6^

In Ghana, the Community-based Health Planning and Services (CHPS) is one of such programmes which aim at taking healthcare to the door-steps of Ghanaians. In this programme, CBVs assist community health officers (CHOs) in health promotion activities, vital event recording, community mobilization, disease surveillance and basic health service delivery.^6-8^ Though these volunteers maybe a group with one purpose, their interests and reasons for volunteering may vary according to their expectations of benefits and sociocultural context of the activity.^9^ Though CBVs extensively complement the health system to extend health services to rural communities in Ghana, studies that have investigated their motivation for volunteering have only been conducted in the northern regions of Ghana.^10^

Dil et al found the love for community as the main motivating factor among community-based volunteers in one of the studies in northern Ghana. Also, they were found to be selfless because the community selected them. However, there were disincentives such as incorrect community perceptions of volunteers, problems with transportation and equipment and the lack of payment for ad hoc tasks such as national immunization days.^10^ Also, it was found that, beyond the love for community-based volunteers in the Kassena-Nankana districts of Upper East region, incentives such as raincoats, torch lights and wellington boots were the mechanisms used to retain and sustain volunteer activities.^11^ Also, in Savelugu, though volunteers love their communities, they were also motivated by community recognition of their services. However, the researchers found that the absence of regular pay and non-monetary rewards such as T-shirts, cutlasses, soap and free medical services were disincentives to volunteers.^12^

Whilst motivation of volunteers in the northern regions of Ghana are considerably known, there is a dearth of evidenced-based study on motivation for volunteering in other parts of the country. We carried out this study to examine the motivation of volunteers working on health-related activities in the middle part of Ghana. Considering the contribution of volunteers in Ghana healthcare delivery systems, gaining understanding of volunteer motivations in other parts of Ghana is important in order for healthcare managers to develop a holistic and effective volunteer recruitment and retention strategies.

### Theories of Volunteer Motivation

A traditional understanding of volunteers motivation is based on the principles of altruism or selflessness^13,14^ where the primary motive for offering voluntary services is the desire to assist others. Contrary to this, volunteers may also offer voluntary services to directly or indirectly benefit themselves and/or their relatives.^15^ Similarly, the Unidimensional Model of Motivation posits that, volunteers are motivated by both altruistic and egoistic motives.^16^ The two-dimensional model on the other hand argues that, the altruistic and egoistic motives are distinct.^17^ The egoistic motives are related to the attainment of tangible rewards such as career-related benefits. Volunteers motivated by egoistic motives engage in volunteer service to improve their own welfare. However, individuals motivated by altruistic motives engage in volunteerism with the purpose of improving the welfare of others. Likewise, extrinsic and intrinsic theories of volunteer motivation are also two-dimensional. Intrinsic motivation is inherently satisfying and volunteers with this type of motivation engage in volunteerism because of inherent satisfaction.^18^ Activities that are extrinsically motivated, however, are performed because of the external outcome that they yield.

The three-dimensional model of motivations divides volunteer motivation into altruistic, material, and social dimensions. Largely, researchers have accepted the three categories and their contents as justification for volunteer motivation. While material motives are derived from the desire for material rewards, social motives also appeal to social perception as motivating factors for examples prestige and recognition.^19^

The Functional Motivation Theory also analyses a broader range of volunteer motivation. The theory is based on two basic principles: (*a*) that individuals engage in purposeful activities to fulfil certain goals and (*b*) that individuals perform the same activities to serve different psychological functions.^20^ The underlying principle here is that volunteers engage in volunteerism because it fulfils certain psychological needs. In view of this, volunteers who may be engaged in the same activities may have different reasons or motives for doing so. The theory implies that individuals will begin and continue to volunteer as long as the activity matches and fulfils the individual’s motivational concerns.^21^

The functional motivation theory identified six broad functions served by volunteering which falls under the following categories such as value, understanding, social, career, protective and enhancement. The value type of motivation is related to the opportunities to express one’s values associated with altruistic and humanitarian concerns for others. The understanding motive deals with opportunities for new learning experiences, to exercise one’s knowledge, skills and abilities. The social factors of volunteer motivation are about the opportunities to be with one’s friends or to engage in an activity viewed favourably important by others. Also, the career is about the experiences that may be obtained from participation in volunteer work. Other function that volunteerism serve, is protective, thus reducing guilt over being more fortunate than others and addressing one’s own personal problems. Finally, the enhancement function is to satisfy the ego for growth and development.^20^

Another principle of volunteer motivation is Abraham Maslow’s Hierarchy of Needs theory. In this context, it is argued that, volunteers have needs that are met through activities that best satisfies those needs. As Maslow postulated, motivation is driven by the existence of unsatisfied needs.^22,23^ The five levels of needs are survival needs (food, cloth, shelter), safety needs (the need to be protected), love, affection, belonging needs, esteem needs (status within a set up) and finally self-actualization needs (personal growth and renewal). It is important to note that, esteem needs may be classified as internal or external. Internal esteem needs are related to self-esteem and achievement. In effect, volunteers with this kind of motivation are affiliated with duties that are very task orientated, where volunteers work to benefit others. External esteem needs are those that relate to social status and recognition and these are usually met through leadership roles. Also, volunteers who are motivated by social needs are looking for experiences that will allow them to interact with others. Their motivation is based on the need to develop friendships, to belong to some group or organization and the need to give and receive love.

The theory of motivation which is adopted by this study is the Social Exchange Theory. The Social Exchange Theory was formally advanced in the works of sociologists George Homans and Peter Blau.^24-26^ Homans and Blau portray individuals as strategic actors who use the resources they have at their disposal in an effort to optimize their rewards. Thus, they contend that individuals are motivated to act not on the basis of tradition, unconscious drives or some type of structural imperative, but rather on the basis of rational considerations thus weighing the consequences of alternative lines of conduct in terms of the profit they will likely generate.^24-26^

The exchange framework is built upon the combination of the central tenets of behaviourism and elementary economics where human behaviour is envisaged as a function of its pay-off. The framework is primarily concerned with the factors that mediate the formation, maintenance, and breakdown of exchange relationships and the dynamics within them. Embedded within the exchange framework are core assumptions about the nature of individuals and about the nature of relationships.^26^ Homan’s five core propositions of human behaviour in formal or informal interaction is relevant to this study as follows; (1) Success proposition states that behavior that creates positive outcomes/reward is likely to be repeated or continued. (2) The stimulus proposition states that if an individual’s behavior is rewarded in the past, the individual is more like continue the previous behavior. (3) The value proposition states that if the result of an action is considered valuable to the individual, it is more likely for that behavior to occur. (4) The deprivation-satiation proposition believes that if an individual has received the same reward several times, the value of that reward will diminish. (5) The aggressive approval proposition states that when an individual’s action does not receive the reward expected or receives punishment that was not expected, the individual is more like to perform aggressive behavior. Also an individual who receive more reward than expected or do not receive anticipated punishment will be happy and will behave approvingly. Individuals are rational beings and within the limitations of the information that they possess, they calculate rewards and costs and consider alternatives before acting.^26^

Volunteers in the study could be volunteering because there is good enough evidence to show that they will receive benefits and rewards. For example some of the personal benefits volunteers have received in their previous volunteering activities include, honorarium (monetary incentives), rain coats, bicycles and T-shirts.^27,28^ Also, volunteers at the study site may be volunteering because stakeholders who engage them, pay extra-duty allowance for example national programmes such as National Immunization.

However, it is important to note that, not all volunteers in the study site may be volunteering because of material benefits. Some could be offering voluntary services because of the desire and the need to help their communities. In spite of this, the Social Exchange Theory is the most suitable for this study because, whatever the motive for volunteering is, it is largely the outcome of some rational considerations as espoused in all the theories of volunteer motivation explained above.

## Methods

### Study Design

A sequential mix-method cross-sectional design employing both quantitative and qualitative techniques were used to examine the motivation and retention among community-based volunteers in the study area between December 2014 and February 2015.

### Study Setting

This study was conducted in the Kintampo North Municipality (KNM) and Kintampo South District (KSD) in the Brong-Ahafo Region of Ghana. The study area is located within the forest-savannah, transitional ecological zone in central Ghana. The study site covers an area of 7162 km^2^ with a resident population of approximately 150 615 in about 157 communities as at 2014.^29^ The study setting is largely rural and subsistence farming is the major occupation. There are 2 referral hospitals each in KNM and KSD. The rest of the health facilities are made up of 12 health centres and 30 CHPS compounds^30^ in the rural communities. Under the CHPS framework community-based volunteers support CHOs in the study site in provision of health service to rural communities. These volunteers receive one to two weeks of training on primary healthcare, hygiene and treatment of minor ailments, family planning, sexually transmitted infections and other endemic diseases. This is done to prepare them adequately for community activities in their respective communities. They are also trained for specific health programmmes as and when the need arise. The volunteers are thus very important in the implementation of the CHPS and extending primary healthcare in Ghana. The Kintampo Health Research Centre (KHRC) operates in the study site and runs the Kintampo Health and Demographic Surveillance System (KHDSS), which provide core updates on pregnancies, births, deaths and migrations (in and out) every 6 months.^31^ In addition to designated field staff who collect data, the KHDSS also relies on these volunteers for updates as they occur in real time.

### Study Procedures

*Sampling Technique and Sample Size:* There were 410 CBVs in the study site; 226 located in KNM area and 184 in the KSD area. The sample size was based on the assumption that 50% of the volunteers at the study site were willing to volunteer. With a 95% CI and a power of 80%, a sample size of 205 CBVs were required for the cross-sectional survey. Out of the 205 CBVs, 113 were randomly selected from KNM, and the remaining 92 from KSD. This was based on proportional allocation of participants according to the number of CBVs in each locality. Simple random sampling was used in the selection of respondents from each of the districts.

*Data Collection Methods:* The data collection methods for this study were triangulated for validation. We used both quantitative (surveys) and qualitative in-depth interviews (IDI), focus group discussion (FGD) approaches for collecting data on reasons for volunteering, incentives received by volunteers (monetary and non-monetary) and volunteers’ satisfaction. The results of the quantitative and qualitative approaches were compared for corroboration and points of divergence.

### Quantitative Approach

The cross-sectional survey was carried out among 205 selected volunteers in KNM and KSD of Ghana between December, 2014 and February, 2015. Experienced field assistants were recruited and trained for data collection for the survey. They were trained on technical content, survey techniques and ethical procedures. During the training, survey questions were read out, explained and translated into “Twi” which is the common language spoken at the study site. In the survey, the pretested paper-based questionnaire was administered by trained research assistants in either English or Twi. Each interview was conducted within 30–60 minutes. The questionnaire explored the socio-demographic characteristics of respondents, their reasons for volunteering, incentives received as volunteers (monetary and non-monetary), and services provided as volunteers and their satisfaction levels as volunteers. The questions were close-ended.

### Qualitative Approach

Using the principle of phenomenology, the survey was followed with IDIs and FGDs to further explore important themes from the quantitative results. The phenomenological approach uses data collection methods such as interviews to understand the meaning participants place on the phenomenon being studied. In this study, we relied on the participants’ own perspectives to provide insight into their motivations for volunteering.

### In-depth Interviews

Twelve IDIs were conducted in 4 communities (2 from KNM and KSD each) among opinion leaders, health staff at CHPS compounds and public health officials at the health directorates. In each district, one of the communities was selected close to the referral health facility and the other relatively far away from the referral health facility. One chief, 2 assemblymen and 1 unit committee chairman were interviewed to explore the role of the community influence on retention among volunteers. At the health directorate, 2 directors of health services, 1 public health nurse and a disease control officer who double as volunteers’ coordinator were interviewed to further understand motivation for retention among volunteers. Also, 3 CHOs and a Health Assistant were interviewed since they work directly with the volunteers and understand their experiences. The IDIs explored themes on the selection of volunteers in the communities, work conditions and incentives received as volunteers (monetary and non-monetary), their satisfaction as volunteers, issues on local institution and community support as well as issues to consider in motivating volunteers.

### Focus Groups Discussions

Two FGDs were done with selected volunteers. Based on the outcome of the survey, 15 volunteers (9 from KSD and 6 from KNM) who took part in the survey were purposively selected for FGDs. In each group, there were discussants who reported satisfaction or dissatisfaction as volunteers during the survey. The two categories of respondents were put together to understand the diversity in their experiences and preferences, whilst comparing what they have to say in a focus group. The FGDs further explored themes on respondents reasons for volunteering, incentives received as volunteers (monetary and non-monetary), their satisfaction as volunteers, issues on local institution and community support as well as issues to consider in motivating volunteers.

### Data Management and Analysis

All completed questionnaires were manually checked for completeness and logged for easy access. Independent double data entry with verification was carried out in Microsoft SQL. Data were analyzed using Stata 12 (College Station, Texas, 7845, USA). Study variables were described using frequencies and percentages. Chi-square test was used in a univariate analysis to test for association between volunteers’ satisfaction and socio-demographic, monetary and non-monetary incentives. Factors found to be associated (at *P* value < .05) with volunteers’ satisfaction in the chi-squared test was included in a logistic regression model in a multivariate analysis to identify significant predictors of volunteers’ satisfaction at (*P* < .05). Two sample independent *t* test was used to test for difference in mean (average) ages among volunteers who were not satisfied and satisfied. The findings of the survey and qualitative interviews are triangulated. FGDs and IDIs were audio-recorded and transcribed verbatim. Records of happenings during interview and discussion sessions was kept and incorporated into the analysis to enhance interpretation of data. Interview transcripts were imported into Nvivo 8 and major themes of interest were developed taking into consideration the objectives of the study. The data were coded according to the themes and summarized as narratives to complement the quantitative findings.

## Results

### Socio-demographic Characteristics of Respondents

Majority (84.9%) of the volunteers were males, 40.5% of them were 50 years and above; over 95% have had at least middle/junior high school education. Majority of respondents were married (76.6%) and Christians (69.8%) (Table 1).

**Table 1:** Socio-demographic Characteristics of Respondents (N = 205)

**Characteristics**	**No. of Respondents**	**Percent**
Gender		
Male	174	84.9
Female	31	15.1
Age groups		
20-24	10	4.9
25-29	16	7.8
30-34	25	12.2
35-39	26	12.7
40-44	24	11.7
45-49	21	10.2
50 and above	83	40.5
Educational background		
No formal education	4	2.0
Primary	4	2.0
Middle school/junior high school	121	59.0
Technical /commercial/senior high school	67	32.6
Polytechnics/nursing /teacher training	4	2.0
University	5	2.4
Marital status		
Married	157	76.6
Living together	13	6.3
Widow	5	2.4
Divorced/separated	8	3.9
Single	22	10.7
Religion		
Christian	143	69.8
Muslim	48	23.4
Other	14	6.8
Ethnicity		
Akan	61	29.8
Mo	34	16.6
Dagarti, Frafra, Kusasi	31	15.1
Gonja, Dagomba, Mamprusi	32	15.6
Other	47	22.9

### Volunteer Motivation

### Reasons for Volunteering

Personal interest (32.7%), community leaders’ selection of volunteers (30.2%) and significance of the work for health services (14.6%) were the main reasons given by respondents for volunteering (Table 2). Also, majority (68.3%) of the respondents continue to offer voluntary services because of the desire to help others whilst (15.1%) indicated satisfaction accrued from helping others. The reasons for volunteer’s retention were corroborated by this response during IDI and FGD sessions:

**Table 2:** Reasons for Volunteering (N = 205)

	**No. of Respondents **	**Percent**
Reasons		
Personal interest	67	32.7
Community leaders selection	62	30.2
Significance of the work for health services	30	14.6
Desire to help community	15	7.3
Work content	8	3.9
Acquisition of knowledge	8	3.9
Family suggestion	5	2.4
Familiarity with community	4	2.0
Other	4	2.0
Work condition	2	1.0
Motivation to continue as a volunteer		
Desire to help others	140	68.3
Satisfaction from helping others	31	15.1
Acquisition of knowledge/learn	12	5.9
Future employment	8	3.9
Social recognition/prestige	7	3.4
Other	7	3.4

*“The recognition is the personal interest. When people volunteer, they become leaders and take advantage of that opportunity to aspire for other positions like the assemblyman. Assemblymen are elected, so if volunteers are active in the community, everybody gets to knows them, so when they contest election as an assemblyman, they are likely to win”* (IDI, Director of Health Services).

### Incentives Received by Volunteers

All the volunteers alluded that they were not paid for their job. Volunteers emphasized that as their titles connotes, their work is purely voluntary as expressed in this response:

“*It is purely voluntary. I think the work we are doing is just to help so that if someone comes from the Ghana health Service or somewhere and ask for the assistance of a volunteer, I could assist. If after assisting for the work to be done the person gives me something I could take it but we are not paid”* (Volunteer 8, FGD, KSD).

Though the volunteers were not paid, majority of them receive monetary incentives such as per diem 101 (49.3%) when they travel for training or workshop outside their communities and extra duty allowance 182 (88.8%) when they are engaged in activities that require additional responsibilities (Table 3), as justified during FGD sessions with volunteers:

**Table 3:** Monetary and Non-monetary Incentives (N = 205)

	**No. of Respondents**	**Percent**
Monetary incentives		
Extra duty allowance	182	88.8
Per diem	101	49.3
Monthly honorarium	0	0.0
Non-monetary incentive		
Training	144	70.2
Food during training	108	52.7
Means of transport	96	46.8
Uniform, backpacks, cap, bicycle	93	45.4
Discount medicine, free ticket for care	6	2.9
Subsidized accommodation	2	1.0
Free accommodation	1	0.5
Food ration/meals	1	0.5

“*It is true the work is voluntary but I have two wives with twelve children and I left them at home to come and sit here. By the time I leave here, I wouldn’t get my “by-day” money to buy plantain and cocoyam for my family. I am listening to what we are discussing so that I can go and help my community. I have wasted my time because I couldn’t go for by-day [work and be paid at the end of the day]. Whilst I sit here others have gone to their farms or for “by-day” but I have left my work and I am sitting here so there should be some compensation” (Volunteer 4, FGD, KSD).*

In relation to the monetary compensation, some of the volunteers stopped working because their expectations of receiving financial rewards were not forthcoming as explained in the response below.

“*One volunteer told me that he thought the man (Community Heath Officer) was sitting on their money so he stopped. He said it was my senior colleague who came to talk to him to come and help because the community people have accepted him. Sometimes they do think that some people have been sitting on their money. Although the thing is voluntary, they think there is some special kind of money for them” (IDI with Community Heath Officer, KNM).*

Training (70.2%) is a key non-monetary incentive among volunteers (Table 3). Other non-monetary incentives that serve as motivation among volunteers include uniforms, backpacks, caps and bicycles (45.4%) and food (52.7%) during training (Table 3).

The response below demonstrates a typical non-monetary incentive for volunteer motivation and retention.

“*Nobody elected me as a volunteer. My uncle was a volunteer and I saw that they were given bicycles which I needed at that time so I decided to be a volunteer”* (Volunteer 4, FGD, KNM).

Also, other important facilitators of retention among volunteers are community recognition and prestige and the preferential treatment given volunteers at health facilities. The excerpt below supports this finding.

“*I don’t join the long queue when I go to the hospital. I always get treatment before most of the people I meet at the hospital all because of the voluntary work I do. I learned that you reap what you sow; that is why I will continue to work as a volunteer” (Volunteer 3, FGD, KNM).*

The response below also corroborates this finding:

“*It is a prestige for volunteers in their community; they call them ‘doctors’ so they feel proud to be working with the health people. Because they are volunteers they are given preferential treatment. If you were the one, how will you feel?”* (IDI, Director of Health Services).

### Services Provided and Volunteers Motivation

Volunteers mainly assist with the provision of child health services (80.5%), referral services (53.2%) and care for new born (50.7%) (Table 4). Though volunteers are respected and recognized, they are also motivated to help in extending these services as a way of reducing the disease burden of their communities. Their involvement as a form of community participation also facilitates the provision of these services as elaborated in the response below.

**Table 4:** Services Provided by Volunteers (N = 205)

**Services Volunteers Assist in Providing**	**No. of respondents**	**Percent**
Child health	165	80.5
Referral	109	53.2
Care for new born	104	50.7
Family planning	49	23.9

“*I have volunteered because children are affected with diseases such as polio, measles and the likes and doctors come to give them injections. If a native of the community is part of them, children or the community members will not be scared when they come for the injection; they happily come so you help the strangers for you to work. That is why I have volunteered”* (Volunteer 4, FGD, KSD).

This finding was substantiated by healthcare provider *“What they (volunteers) do is that, for instance when there is NID, they drop it in the children’s mouth. That when they do, they are paid”* (IDI with Community Heath Officer, KSM)*.*

### Volunteers Satisfaction

Overall, majority (87.8%) of volunteers were satisfied with their work (Table 5) because of the recognition and preferential treatment they receive as volunteers as explained in the excerpts below.

**Table 5:** Volunteers Work Satisfaction With the Work Component

**Volunteers Work Component**		**Satisfied**	**Dissatisfied**
Level of health services provided by volunteers	n	198	7.0
	%	96.6	3.4
Non-monetary incentives	n	91	114
	%	44.4	55.6
Working hours	n	167	38
	%	81.4	18.6
Location of work	n	161	44
	%	78.5	21.5
Training	n	193	12
	%	94.1	5.9
Supplies for work	n	148	57
	%	72.2	27.8
Community members support	n	84	121
	%	41.0	59.0
Health committee support	n	66	139
	%	32.2	67.8
Supervision received	n	166	39
	%	81.0	19.0
Overall satisfaction	n	180	25
	%	87.8	12.2

“*I am satisfied because when health workers visit the community, I am the first person they look for and this makes me proud and popular. Sometimes I also feel I am a health worker and I am respected by people in my community and visitors as well”* (Volunteer 2, FGD, KSD).

Also, majority (94.1%) of the volunteers were satisfied because of the training they receive as volunteers (Table 5) as summarized in the response below.

*“Though the work is voluntary, most of us learn a lot of things from it which helps us in our life; we get a lot of training. We have been here (Health Directorate) on several occasions; the hospital also calls us; training in how to handle children, cholera and a lot of thing; because you have volunteered, the health people call you” (Volunteer 9, FGD, KSD).*

However, some of the volunteers (27.8%) were dissatisfied because they feel there is discrimination in the distribution of items meant for volunteers (Table 5) as demonstrated in the response which follows:

“*For instance, you give someone in Kintampo North a bicycle but you didn’t give the person in the south and the person will have to walk to do the work. If it happens this way, it means there is cheating and once there is cheating I have to stop”* (Volunteer 9, FGD, KSD).

Similarly, majority (59.0%) and (67.8%) of the volunteers were dissatisfied with the community members’ and Community Health Committees’ support respectively (Table 5). Community members do not support volunteers with any form of incentives because they perceive volunteers are paid by Ghana Health Service and programme managers who engage them.

*“We all know the work is voluntary but someone could ask wouldn’t you have stopped if there is no pay? You take pay! Some people say very unpleasant things about volunteers. They say that we take money, whilst we don’t get anything. If it continues this way, I could think of stopping for another person to come and see if there is money in it” (Volunteer 2, FGD, KSD).*

### Chi-Square Test for Volunteers’ Satisfaction and Demographic, Monetary and Non-monetary Incentives Variables

Table 6 presents a chi-square test to test the association between volunteers’ satisfaction and socio-demographic variables, monitory and non-monetary incentives. Only per diem showed a significant association with volunteers’ satisfaction with a *P* value of .046 (Table 6).

**Table 6:** Association Between Demographic, Monetary and Non-monetary Incentives Variables Volunteers’ Satisfaction

**Variables**	**Volunteers’ Satisfaction** **No. (%)**		***P*** ** Value**
	**Not Satisfied**	**Satisfied**	
**Demographic Characteristics**			
Gender			
Male	22 (88)	3 (12)	.642
Female	152 (84.4)	28 (15.6)	
Educational level			
None	0 (0.0)	4 (100.0)	.270
Primary school	0 (0.0)	4 (100.0)	
Middle/JHS	12 (9.9)	109 (90.1)	
Technical/commercial	10 (15.0)	57 (85.0)	
Polytechnic/nursing/teacher training	1 (15.0)	3 (75.0)	
University	2 (40.0)	3 (60.0)	
Marital status			
Married	17 (10.8)	140 (89.2)	.692
Living together	2 (15.4)	11 (84.6)	
Widow	0 (0.0)	5 (100.0)	
Divorced	1 (25.0)	3 (75.0)	
Separated	1 (25.0)	3 (75.0)	
Single, unmarried	4 (18.2)	18 (81.8)	
Religion			
Christian	19 (13.3)	124 (86.7)	.716
Muslim	6 (12.5)	42 (87.5)	
Traditional African	0 (0.0)	5 (100.0)	
Pagan (No religion)	0 (0.0)	6 (100.0)	
Other	0 (0.0)	3 (100.0)	
**Monetary Incentives**			
Per diem			
Yes	17 (16.8)	84 (83.2)	.046
No	8 (7.7)	96 (92.3)	
Payment for extra duty activities			
Yes	20 (5.8)	162 (94.2)	
No	5 (2.7)	18 (78.3)	.138
**Non-monetary Incentives**			
Uniforms, backpacks, cap, etc			
Yes	13 (14.0)	80 (86.0)	.524
No	12 (10.7)	100 (89.3)	
Discount on medicine			
Yes	1 (16.7)	5 (83.3)	.734
No	24 (12.1)	175 (87.9)	
Training			
Yes	15 (10.4)	129 (89.6)	.232
No	10 (16.4)	51 (83.6)	
Food rationing			
Yes	0 (0.0)	1 (100)	.709
No	25 (12.3)	179 (87.7)	
Subsidized accommodation			
Yes	0 (0.0)	2 (100)	.596
No	25 (12.3)	178 (87.7)	
Transport			
Yes	13 (13.5)	83 (86.5)	.580
No	12 (11.0)	97 (89.0)	
Food during training			
Yes	11 (10.2)	97 (89.8)	.353
No	14 (14.4)	83 (85.6)	

Abbreviation: JHS, Junior High School

### Relationship Between Satisfaction and Age

Table 7 presents two sample independent t-test for differences in mean (average) ages among the two groups (volunteers who were not satisfied and satisfied). The difference in mean ages was 3.124 with a *P* value of .265 (Table 7).

**Table 7:** Two Sample Independent *T* Test for Satisfaction and Age

**Group**	**Mean**	**SD**	**95% CI**
Satisfied	45.44	12.83	43.60-47.33
Not satisfied	42.32	15.04	36.11-48.53

Abbreviation: SD, standard deviation.

### Logistic Regression for Per Diem

Logistic regression was computed for per diem after it was found to have a significant association with volunteers’ satisfaction. In the logistic regression for per diem, volunteers who did not receive per diem were 2.4 times more likely to be satisfied compared to those who received per diem but there was a weak evidence (*P* = .051) to support this difference (Table 8).

**Table 8:** Logistic Regression for Per Diem

**Variable**	**OR**	***P*** ** Value**	**95% CI**
Per diem			
Volunteers who receive per diem	ref		
Volunteers who do not receive per diem	2.42	.051	0.99-5.91

## Discussion

Considering the contribution of community volunteers in healthcare delivery systems, gaining understanding of volunteer motivations is important in order for healthcare managers to develop effective volunteer recruitment and retention strategies. This study investigated the motivation and retention of volunteers working on health-related activities in the middle part of Ghana.

Our study suggests that, volunteers were motivated by their personal interest to serve their communities. Being volunteers in these settings comes with community recognition and popularity. The recognition and popularity give volunteers an advantage over other contenders in the event of contesting for community-based positions like district assembly member. Again, it provides them the opportunity to further their education particular in health training institutions because of their involvement in health service delivery in their communities. Our study yielded similar results with a study in Nepal which showed that volunteers see volunteering as a medium to fulfil their personal goals of working. Volunteer work is therefore perceived as useful and valuable as it helps them fulfil their personal interest.^32^

Also, volunteers were motivated to serve their communities because it is deemed a privilege to be selected and entrusted with the responsibility of supporting healthcare provision in the community by community leaders. The social structure is such that each community is headed by a chief. The chief and his elders in consultation with the Health Directorates select the volunteers in their respective communities.^7^ This finding is similar to findings in the northern region of Ghana, where volunteers were motivated to offer services because of the love for their communities and their selection by community.^10^

Contrary to the egoistic principle of motivation,^18^ our study found that, volunteers desired to assist in improving the health and wellbeing of their people. Most of the volunteers in the study belong to the same ethnic group of the people they serve, as such feel a sense of belongingness and hence a need to help their people. This finding is comparable to results from similar studies in the upper east and northern regions of Ghana. In these studies, it was found that, volunteers were motivated to volunteer because of their love for their communities.^10,12^ Also, volunteers in mother-to-mother support groups in rural Ghana were found to be motivated by their desire to help other mothers have healthy children.^33,34^ Also, volunteers felt compelled to volunteer because of the need to complement the staff strength of the local health system to improve on the health of their community members. Volunteers assist health staff at the CHPS compounds and outreach programmes to provide health services. This finding is in line with the goal of Primary Healthcare in Ghana, which emphasizes the right and duty of community members to participate individually or collectively in the planning and implementation of their healthcare.^8,35,36^

In our study, there was a weak evidence to suggest that volunteers were motivated by per diem provided to them. They were however motivated by extra duty allowance. This is likely because per diem were paid to volunteers who traveled out of their communities to participate in workshops or training and used for cost incurred on accommodation, meals and other incidentals; therefore unlikely to gain from it. Per diems were therefore unlikely to be an income to volunteers.^37^ Unlike per diem, extra duty allowance were likely to be considered as an income by the volunteers since they were given to volunteers for their personal expense when they performed additional activities within their communities.^38^ This finding is consistent with the findings in other parts of Ghana^10,12^ and in Zambia where volunteers in service programmes receive transport refunds when they attend seminars and training but are motivated by allowances they receive for their extra duties.^39^ The volunteers were motivated by monetary income probably because community-based volunteers in rural settings are less likely to be formally employed and may require monetary incentives to support their household income.^40^ It is however unclear how volunteers will or do respond to calls for volunteerism in the absence of such monetary incentive. Further research is required to examine the behavior of community-based volunteers in the absence of monetary incentives or when monetary incentives dwindle overtime in the same program.

The finding on training as a non-monetary incentive is consistent with the postulations of the Functional motivation theories.^41^ These theories focus on the opportunities for new learning experiences, exercising one’s knowledge, skills and abilities as volunteer motivation for offering services. In this study some volunteers were motivated to volunteer because they benefit from skills training, which were used in their personal lives. Also, the result is in line with Bhattacharyya’s argument that learning new skills is one of the main reasons for volunteering. Bhattacharyya^6^ argued that apart from the opportunity to learn new skills and receive education, training enables volunteers to interact with higher levels of professional staff and gain recognition that they will not obtain if they were not volunteers. Similarly in Nepal, further training enabled volunteers to identify causes and treatment of night blindness and to recognize fast breathing as a major sign of acute respiratory infection.^42^ The implication is that their ability to offer treatment augmented their motivation to volunteer. Also, continuous training gave village health helpers in Kenya enough motivation to continue working even without financial support.^43^ Also, food served during training were sometimes considered “continental” (foods that were not traditional to the volunteers eg, “Jollof” rice) in nature and much appreciated by volunteers as such a good source of motivation among them.

Volunteers were mainly satisfied because of the recognition and preferential treatment they receive at health facilities and by healthcare managers. Though they were not trained health workers they felt they were ‘doctors’ by their association with health workers. In a similar study in Savelugu, in the northern region of Ghana, volunteers cited popularity and recognition by the community as factors that sustain their interest in voluntary work.^12^ In Mexico, it was found that local drug sellers were seen as doctors (Casi Como doctor). People present their health complaints and describe their symptoms, expecting them to diagnose their illnesses and to recommend treatment.^44^

Community-based volunteers’ dissatisfaction with community members’ and community health committees’ support contravene the purpose of the CHPS which is a community-based service delivery that places emphasis on a better cooperation between the health system and households, community leaders and social groups. This cooperation is aimed at addressing the demand side of service provision and in recognizing that households are the primary producers of health.^35^ Also some volunteers feel cheated for not given some incentives such as bicycles because they were oblivious of the fact that they work on vertical programmes with different incentive packages and support systems. This finding resonates with the aggressive/approval proposition of the Social Exchange Theory which discusses when emotions occur due to different reward situations.^26^

Findings from this study gives weight to the social exchange theory particularly the value and success proposition which emphasize the motivation for individual to continue to engage in a behavior as long as the reward from such a behavior is of value to the person.^26^ Volunteers in this study value the recognition, popularity, educational/learning opportunities, fulfillment of helping the community, per diem and extra-duty allowance as valuable rewards they derive from offering voluntary service hence their motivation to continue to volunteer. Similarly, on the success proposition volunteers in this study had indeed been rewarded some form of monetary and non-monetary incentives hence the more they continued to volunteer. It is therefore not surprising that some volunteers whose expectation were not met with regards to reward they envisaged to derive before volunteering felt cheated and express apathy in the performance of their duties. In effect the volunteers were rational beings and within the limitations of the information that they possess calculated the value of the rewards and costs before volunteering.

However, the principles of social exchange do not necessarily apply to all exchanges because of restrictions created by social structure and roles. Social structures have the power to influence human behavior. Social exchange might be occurring at the cognitive level but such exchanges occur because they are defined by the social structure in which we find ourselves.^45^ In this study, some of the volunteers who were selected by community leaders to volunteers might be doing so not necessarily because of the prestige of being selected but have no choice than to accept the responsibility.

There were other interesting findings on the demographic characteristics of the volunteers. Volunteers were mainly males because Ghana is still largely a patriarchal society where men continue to dominate in most spheres of life.^46-50^ In this study, men dominated in volunteering largely because the decision of women to engage in pro-social behaviours such as volunteering is subjected to the acceptance or rejection of men who were mostly gate keepers. This finding is in keeping with the dominant status model, which emphasize less participation for minorities/marginalized because of their less prevalent social positions in a sociocultural context.^51^ However, a study in Malawi showed that all volunteers were females because volunteers were required to visit the sick at the hospital or home and assist with household chores such as sweeping, bathing patients and helping out with cooking. These roles are generally female oriented within the sociocultural arrangement of Malawi.^52^ Similarly, in Kenya, it was observed that, volunteers are mainly women because, they see volunteerism as a critical part of a woman’s responsibility to the society.^53^ Also, in Nepal, volunteers are females because, they assist in referring pregnant women in labour.^32^

The educational background of volunteers is consistent with demographic trends at the study site because most of the volunteers were males and are likely to have acquired basic school education. A study has shown that a higher percentage of females have no education as compared to males at the study site and disparity in gender becomes more apparent as one climbs the academic ladder with less females making it to junior and senior high schools. This is due to teenage pregnancy and sociocultural arrangement that gives preference to male education over females.^54^ Also, majority of respondents have had primary school education and above because to be a volunteer one should be able to read and write.

Our study findings indicate that majority of the volunteers were married. Also, per the social construct of Ghana, married people are largely regarded as responsible and capable of taking up positions such as a community-based volunteer. Studies have documented that, it is only when a person is married, that he/she is taken seriously in social deliberations in the sociocultural arrangement in Ghana.^46,55^ Volunteers at the study site, are engaged in community mobilization and health education as such “being responsible” is an important attribute volunteers must have to enhance their acceptance for the performance of their duties.

Furthermore, the provision of some basic health services is in line with the goals of Primary Healthcare where volunteers support health workers in extending health services to people in rural communities. In 2009, KHRC in Ghana trained some volunteers in its research area to identify pregnant women in their communities, visit them during pregnancy and in the first week of life to promote essential newborn-care practices such as weighing and assessing babies for danger signs and make referral where necessary.^28^ Their source of motivation hinges on the fact that they are respected and regarded as ‘doctors’ in their communities for the services they provide.

## Conclusion

Community-based volunteers’ motivation and retention were influenced by their personal interest in the form of recognition by community members and health workers, community leaders’ selection and other non-monetary incentives. Volunteers were motivated by extra-duty allowance but not per diems paid for the accommodation and feeding when they travel. The behavior of the volunteers in this study is in line with the principles of Social Exchange Theory having been motivated to volunteer on the basis of some benefits that they were likely to derive. The behavior of the volunteers in this study is therefore a function of its pay-off whether monetary or non-monetary. Organizations that engage community volunteers are encouraged to strengthen the selection of volunteers in collaboration with community leaders, and to provide both non-monetary and monetary incentives to motivate volunteers.

## Acknowledgements

The study team expresses their sincere gratitude to the volunteers of KNM and KSD for their participation. We also thank Awurabena Queayeba Dadzie and the field supervisors of the KHDSS for their assistance and support during data collection. We are also grateful to staff of the KHRC for the various roles they played in collecting and processing the data. We also acknowledge KHRC for granting a study leave and sponsoring this Master’s Degree. Our gratitude also goes to the University of Ghana, Accra, Ghana and Carnegie Corporation of New York, USA for granting me support towards my project work and write up of my Master of Philosophy thesis.

## Ethical issues

Ethical clearance was obtained from the Institutional Ethics Committee (FWA 00011103) of the Kintampo Health Research Centre, Kintampo, Ghana. Written informed consent was sought from all study respondents. Participation was voluntary and respondents were informed that they were free to withdraw from participation. They were also assured of the confidentiality of the information they provided.

## Competing interests

Authors declare that they have no competing interests.

## Authors’ contributions

SAA conceived the idea, wrote the proposal, lead the writing of the paper, collected and analyzed the data with the support of a statistician. KPA reviewed the paper and provided technical guideline and support in shaping the paper. KS was my academic supervisor. He was involved in the conception of the idea and supervised the proposal writing and implementation of the project. He reviewed the paper and provided technical guidance and support. MAA contributed to conception of the idea, assisted with data the quantitative data analysis and reviewed the paper. SA reviewed the paper and provided technical guidance and support. EM was the statistician on the project. He assisted in the sample size calculation and data analysis with Stata. EAA assisted with the training of data collectors, supervised data collection and reviewed the paper. AM assisted with the training of data collectors, supervised data collection and reviewed the paper. MLD reviewed the paper. LGF reviewed the paper, assisted in the qualitative data analysis and provided technical support in shaping the paper. SOA provided the overall mentorship and technical support in the conception, design and implementation of the study and the paper writing.

## Authors’ affiliations

^1^Kintampo Health Research Centre, Ghana Health Service, Kintampo, Ghana. ^2^Department of Sociology, School of Social Science, University of Ghana, Accra, Ghana. ^3^Institute of Health Research, University of Health and Allied Sciences, Ho, Ghana.

## Key messages

**Implications for policy makers**

- Volunteer involving organizations should take note of monetary and non-monetary incentives among volunteers to help in their recruitment and retention.
- Attention should be given to community leaders’ selection of volunteers since it gives volunteers traditional authorization and also serve as an important motivation among them.
- Healthcare managers and administrators should encourage the establishment of context-specific community incentive systems to motivate and retain volunteers.
- There is the need for the Ministries of Health and health service managers to establish a general incentive package for volunteers in low resource settings to sustain their interest. Though the work is voluntary, volunteers work in relatively poor communities where the standard of living is relatively low. Incentives in the form of monthly honorarium (monetary) will motivate volunteers to give out their best to complement the health system.

**Implications for the public**

As part of addressing health workforce challenge particularly in developing countries community-based volunteers (CBVs) assist professional health workers to extend healthcare coverage and key health interventions to rural communities and some urban settings. The CBVs at our study site are no exception. However, our findings revealed that these volunteers do not receive salary for services rendered but volunteer to complement the staff strength of the local health system to improve on the health of their community. Understanding motivations of these volunteers is therefore important in order for healthcare managers and community/public to develop effective volunteer recruitment, motivation and retention strategies. Also community members/ public need to be sensitized that, Community-based Volunteers in Ghana are not paid as such they require community support to continue volunteering. The support could be recognition of their services through exemptions from communal labour, words of encouragement and helping volunteers to work on their farms periodically.

## References

[1] World Health Organization. The World Health Report 2006 - working together for health. Gneneva, Switzaland: WHO; 2006.

[2] World Health Organization. Global health workforce shortage to reach 12.9 million in coming decades. http://www.who.int/mediacentre/news/releases/2013/health-workforce-shortage/en/. Published 2013.

[3] Pillinger J. Quality health care and workers on the move. France: Public Services International; 2011.

[4] WHO and UNICEF. Primary Health Care: A Joint Report [on the International Conference on Primary Health Care, Alma-Ata, USSR, 6-12 September 1978]. World Health Organization; 1978.

[5] Spencer MS, Gunter KE, Palmisano G (2010). Community health workers and their value to social work. Soc Work.

[6] Bhattacharyya K. Community health worker incentives and disincentives: how they affect motivation, retention, and sustainability. Arlington, Virginia: Basic Support for Institutionalizing Child Survival Project (BASICS II) for the United States Agency for International Development; 2001.

[7] Awoonor-Williams JK, Feinglass ES, Tobey R, Vaughan-Smith MN, Nyonator FK, Jones TC (2004). Bridging the gap between evidence-based innovation and national health-sector reform in Ghana. Stud Fam Plann.

[8] Binka FN, Nazzar A, Phillips JF (1995). The Navrongo Community Health and Family Planning Project. Stud Fam Plann.

[9] Community Health Workers and Systems. http://www.coregroup.org/our-technical-work/program-learning/community-health-workers. Published 2002.

[10] Dil Y, Strachan D, Cairncross S, Korkor AS, Hill Z (2012). Motivations and challenges of community-based surveillance volunteers in the northern region of Ghana. J Community Health.

[11] Acheampong T. Factors Influencing Sustainability of Community-Based Health Volunteers Activities in the Kassena-Nankana East and West Districts of Northern Ghana. University of Ghana; 2012.

[12] Agyemang D. Incentives and disincentives of community health workers in Ghana. December 29, 2014.

[13] Phillips M (1982). Motivation and expectation in successful volunteerism. J Volunt Action Res.

[14] Rehberg W (2005). Altruistic Individualists: Motivations for International Volunteering Among Young Adults in Switzerland. Voluntas: International Journal of Voluntary and Nonprofit Organizations.

[15] Silverberg Silverberg, KE KE, Backman SJ, Backman KF, Ellis GD (1999). An identification and explication of a typology of public parks and recreation volunteers. World Leisure & Recreation.

[16] Cnaan RA, Goldberg-Glen RS (1991). Measuring Motivation to Volunteer in Human Services. J Appl Behav Sci.

[17] Frisch MB, Gerrard M (1981). Natural helping systems: A survey of Red Cross volunteers. Am J Community Psychol.

[18] Finkelstien MA (2009). Intrinsic vs extrinsic motivational orientations and the volunteer process. Pers Individ Dif.

[19] Widjaja E. Motivation behind volunteerism [CMC Senior Theses]. http://scholarship.claremont.edu/cmc_theses/4/. Published 2010.

[20] Clary EG, Snyder M, Ridge RD (1998). Understanding and assessing the motivations of volunteers: a functional approach. J Pers Soc Psychol.

[21] Clary EG, Snyder M (1999). The motivations to volunteer: theoretical and practical considerations. Curr Dir Psychol Sci.

[22] Maslow AH (1943). A theory of human motivation. Psychol Rev.

[23] Neher A (1991). Maslow’s theory of motivation: a critique. Journal of Humanistic Psychology.

[24] Blau PM. Exchange and Power in Social Life. Transaction Publishers; 1964.

[25] Homans GC (1958). Social Behavior as Exchange. Am J Sociol.

[26] Homans GC. Social behavior: Its elementary forms. New York: Routledge & Kegan Paul; 1961.

[27] Kirkwood BR, Manu A, ten Asbroek AH (2013). Effect of the Newhints home-visits intervention on neonatal mortality rate and care practices in Ghana: a cluster randomised controlled trial. Lancet.

[28] Manu AA. Newhints Home Visits randomised controlled trial: impact on access to care for sick newborns and determinants, facilitators and barriers to this [Thesis]. London School of Hygiene & Tropical Medicine; 2012.

[29] Kintampo Health Research Centre Annual Report 2013/2014 Annual Report. Ghana: Kintampo Health Research Centre Annual Report; 2014.

[30] Kwarteng A, Asante KP, Abokyi L (2015). Provider compliance to artemisinin-based combination therapy at primary health care facilities in the middle belt of Ghana. Malar J.

[31] Nettey OE, Zandoh C, Sulemana A, Adda R, Owusu-Agyei S (2010). Clustering of childhood mortality in the Kintampo Health and Demographic Surveillance System in Ghana. Glob Health Action.

[32] Swechhya B, Kamaraj R (2014). Female Community Health Volunteers Program in Nepal: Perceptions, Attitudes and Experiences on Volunteerism among Female Community Health Volunteers. Int J Interdiscip Multidiscip Stud.

[33] Quinn VJ, Guyon AB, Schubert JW, Stone-Jiménez M, Hainsworth Hainsworth, MD MD, Martin LH (2005). Improving breastfeeding practices on a broad scale at the community level: success stories from Africa and Latin America. Journal of Human Lactation.

[34] SPRING. Ghana: Final Country Report 2014–2017. Arlington VA. Strengthening Partnerships, Results, and Innovations in Nutrition Globally (SPRING) project; 2018. https://www.spring-nutrition.org/sites/default/files/publications/reports/spring_ghana_eop_report.pdf.

[35] Binka F, Aikins M, Sackey OS. In-depth Review of the Community-based Health Planning Services (CHPS) Programme, A report of the Annual Health Sector Review 2009. Ghana: School of Public Health; 2009. http://www.moh.gov.gh/wp-content/uploads/2016/02/CHPS-Review-Report-FINAL-180509.pdf.

[36] MoH MoH (1999). Community-based Health and Planning Services (CHPS) handbook.

[37] How to Use ‘Per Diem’ in a Sentence. https://www.merriam-webster.com/words-at-play/what-does-per-diem-mean. Published 2018.

[38] Special duty and etra duty allowances. http://prb.pmo.govmu.org/English/Documents/PRB%20Reports/extraduty.pdf. Published 2013.

[39] Wilson T. Incentives and volunteerism in Zambia: A review. Research Partnerships Build the Service Field in Africa; 2007:68-84.

[40] Shumba R. Incentives and volunteering: the debate continues. Focus 2007;3(1). http://vosesa.org.za/focus/vol3_no1/index.html?article_2.html~content.

[41] Clary EG, Snyder M, Ridge RD (1998). Understanding and assessing the motivations of volunteers: a functional approach. J Pers Soc Psychol.

[42] Curtale F, Siwakoti B, Lagrosa C, LaRaja M, Guerra R (1995). Improving skills and utilization of community health volunteers in Nepal. Soc Sci Med.

[43] Kaseje DC, Spencer HC, Sempebwa EK (1987). Characteristics and functions of community health workers in Saradidi, Kenya. Ann Trop Med Parasitol.

[44] Logan K. ‘Casi Como Doctor’: Pharmacists and Their Clients in a Mexican Urban Context. In: van der Geest S, Whyte SR. The Context of Medicines in Developing Countries: Studies in Pharmaceutical Anthropology. Dordrecht: Springer; 1988:107-129.

[45] Redmond MV. Social Exchange Theory. English Technical Reports and White Papers. https://lib.dr.iastate.edu/engl_reports/5/. Published 2015.

[46] Nukunya GK. Tradition and Change in Ghana: An Introduction to Sociology. Ghana Universities Press; 2003.

[47] Oppong C. Growing up in Dagbon. Tema, Ghana: Ghana Publishing Corporation; 1973.

[48] Oppong C, Abu K. Seven roles of women: impact of education, migration and employment of Ghanaian mothers. ILO; 1987.

[49] Sossou MA (2006). The meaning of gender equality in Ghana: women’s perceptions of the issues of gender equality: implications for social work education and practice in Ghana. Women in Welfare Education.

[50] Sossou MA (2011). We Do Not Enjoy Equal Political Rights: Ghanaian Women’s Perceptions on Political Participation in Ghana. Sage Open.

[51] Pratto F, Sidanius J, Levin S (2006). Social dominance theory and the dynamics of intergroup relations: Taking stock and looking forward. Eur Rev Soc Psychol.

[52] Mkandawire WC, Muula AS (2005). Motivation of community care givers in a peri-urban area of Blantyre, Malawi. Afr J Health Sci.

[53] Ochieng BM, Kaseje DO, Mala SJ, Mumbo HM, Aila FO, Odera O (2012). Motivational drivers for non-skilled Kenyan community health volunteers. Int J Asian Soc Sci.

[54] Owusu-Agyei S, Nettey OE, Zandoh C (2012). Demographic patterns and trends in Central Ghana: baseline indicators from the Kintampo Health and Demographic Surveillance System. Glob Health Action.

[55] Assimeng M. Social Structure of Ghana: A Study in Persistence and Change. Ghana Publishing Corporation; 1999.

